# Anaphylaxis to dextromethorphan with positive skin testing: a case report

**DOI:** 10.1186/s13223-025-00987-y

**Published:** 2025-10-31

**Authors:** Eman Badawod, Jackie Campbell, Erika Lee

**Affiliations:** 1 Clinical Immunology and Allergy, Department of Internal Medicine, King Abdulaziz University, Jeddah, Saudi Arabia; 2 Drug Allergy Clinic, Sunnybrook Health Sciences Centre, Toronto, ON Canada; 3 Division of Clinical Immunology & Allergy, Department of Medicine, University of Toronto, Toronto, ON Canada

**Keywords:** Anaphylaxis, Dextromethorphan, Drug hypersensitivity, Cough suppressant, Adverse reaction

## Abstract

**Background:**

Dextromethorphan (DM) is a cough suppressant that is widely available in many prescribed and over-the-counter medications. Both immediate and delayed hypersensitivity reactions have been reported following the ingestion of DM. Anaphylaxis to DM is rare, with only three reported cases in the literature. Among these, skin prick testing for DM yielded a positive result in one case, a negative result in another, and was not performed in the third.

**Case presentation:**

We present a rare case of anaphylaxis linked to DM, confirmed by skin testing. The patient experienced a severe allergic reaction after taking Vicks Dayquil Complete®, a common over-the-counter cold and flu medicine that contains acetaminophen, DM, phenylephrine hydrochloride, and guaifenesin. Among these, only DM triggered a positive response on skin testing.

**Conclusion:**

This case highlights an uncommon allergy to a combination cold medicine, with skin testing identifying DM as the cause.

## Background

Dextromethorphan (DM) is a commonly used antitussive medication. It is widely available worldwide on its own or in combination with other active ingredients, such as analgesics, antihistamines and expectorants [1]. DM acts centrally to inhibit the N-methyl-D–aspartate (NMDA) receptors at the medullary cough center [2]. The indications and uses of DM are expanding beyond its use for cough relief. [3] DM is a prodrug that is metabolized in the liver by the CYP2D6 system to dextrorphan, the major active metabolite. [4] Both immediate and delayed hypersensitivity reactions have been reported such as acute generalized exanthematous pustulosis (AGEP) [5]. Only three reports of immediate allergic reactions are described in the literature, and all involve anaphylaxis to DM [6–8].

The exact mechanism for the immediate allergic reactions to DM remains unclear with possible mechanisms including IgE-mediated reactions and direct mast cell activation through MRGPRX2 receptor activation [9].

We describe a case of anaphylaxis to a cough syrup containing DM with subsequent skin testing positive for DM only, suggesting a likely IgE-mediated reaction.

## Case presentation

We evaluated a 31-year-old male for anaphylaxis to an over-the-counter syrup Vicks Dayquil Complete® Cold and Flu, which contains acetaminophen, DM, phenylephrine and guaifenesin. The patient described generalized hives, lip swelling and presyncope, within twenty minutes of ingestion of Vicks Dayquil Complete® Cold and Flu. He was treated with epinephrine, and his symptoms resolved. He reported a similar reaction years ago following the ingestion of an unknown formulation of Benylin®. He has demonstrated tolerance to acetaminophen since the index reaction. He had a known history of exercise-induced asthma, treated with short-acting bronchodilators as needed. He also had a history of allergic rhinitis managed with antihistamines as needed. There was no family history of atopy or drug allergies.

The patient was referred to our drug allergy clinic for cold relief medication allergy. Skin prick testing was performed on the volar aspect of the forearm three times because of inconsistent results (Table 1). Histamine was used as a positive control and glycerinated phenol-saline (sodium chloride 0.9%, glycerin 50%, phenol as preservative 0.4%) was used as a negative control. All medications were tested at full strength without dilution. A wheal diameter of ≥ 3 mm greater than the negative control at 15–20 min was considered positive.

**Table 1:** Skin prick testing results for Dayquil Sinus Liquicaps® (DSL), Benylin Extra Strength All In One Syrup® (BES) and single-entity products

Product/Ingredient (all used undiluted)	Skin prick testing		
	1st	2nd	3rd
Dayquil® Sinus Liquicaps (DSL) (Ingredients: Acetaminophen 325 mg/capsule Phenylephrine HCl 5 mg/capsule	Negative	Negative	Negative
Benylin® Extra Strength All-In-One Syrup (BES) (Ingredients: Acetaminophen 500 mg/15 mL Dextromethorphan Hydrobromide 15 mg/15 mL Guaifenesin 100 mg/15 mL Pseudoephedrine Hydrochloride 30 mg/15 mL	Indeterminate	Negative	Indeterminate
Dextromethorphan syrup 15mg/5mL	Positive	Negative	Positive
Guaifenesin syrup 100mg/5mL	Not performed	Negative	Indeterminate
Histamine (positive control)	Positive	Positive	Positive
Glycerinated phenol-saline: sodium chloride 0.9%, glycerin 50%, phenol as preservative 0.4% (negative control)	Indeterminate	Negative	Negative

A negative result was recorded when there was no observable wheal response. Reactions that did not meet the threshold for positivity (i.e., wheal present but < 3 mm greater than control) were considered indeterminate. Two healthy, unexposed control subjects were tested for sensitivity to DM, and both yielded negative results.

**Fig. 1:**
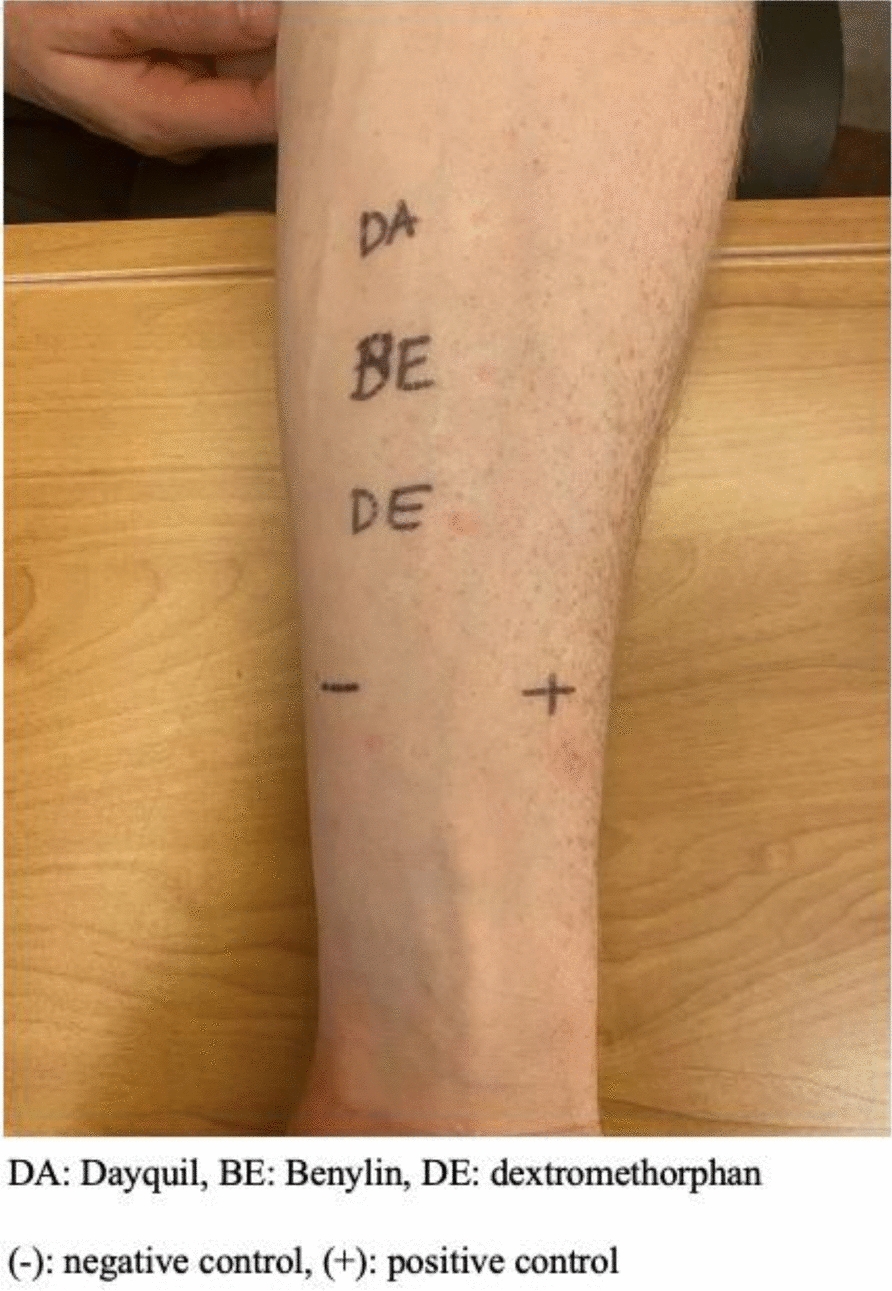
Skin prick test reactions to Dayquil Sinus Liquicaps®, Benylin Extra Strength All-In-One Syrup ®, and dextromethorphan, showing a positive reaction for DM

The first skin prick test was performed using Dayquil Sinus Liquicaps® (DSL), Benylin Extra Strength All-In-One Syrup ® (BES), DM and guaifenesin syrups. DSL, which contains acetaminophen and phenylephrine, was selected as Vicks Dayquil Complete® Cold and Flu was not readily available. BES, which contains acetaminophen, DM guaifenesin and pseudoephedrine hydrochloride, was selected given the patient’s history of a similar reaction to an unknown formulation of Benylin®, and some of these multi-ingredient products contain DM. Test results were negative for DSL, indeterminate for BES and negative control and positive for DM and histamine control (Fig. 1). The second test showed negative results for DSL, BES, negative control, DM and guaifenesin, with a positive histamine control. The third prick test was performed on bilateral arms and revealed negative results for DSL and negative control, was indeterminate for BES and guaifenesin, and positive for DM and histamine control.

As skin prick testing for guaifenesin was indeterminate, unlike the clearly positive result for DM, we discussed options including avoiding guaifenesin or proceeding with a graded oral challenge for further evaluation. Our patient underwent a two-step graded challenge to guaifenesin to a total dose of 200 mg, which he tolerated.

The patient was diagnosed with a type I hypersensitivity reaction to DM on the basis of his clinical history and positive skin prick test for DM syrup. The patient was advised to avoid DM and instructed to verify that there was no DM in any over-the-counter medications he considers taking in the future.

Skin prick testing was performed to evaluate allergic reactions to Dayquil® (DA), Benylin® (BE), and dextromethorphan (DE). A positive reaction was observed for DE, indicated by a raised wheal and erythema. Testing for guaifenesin was conducted twice, with initial results showing no reaction, followed by an indeterminate result in subsequent testing (data not shown). Histamine was used as a positive control, indicated by ( +), and glycerinated phenol-saline served as a negative control, indicated by (-).

## Discussion

DM is a frequently used over-the-counter cough suppressant that is available as a single-entity product or in multiple-ingredient formulations. Allergic reactions have been reported following the ingestion of DM [6–8].

To date, there have been three reports of anaphylaxis after the ingestion of a DM-containing compound. The first reaction was described in a 40-year-old female who experienced the immediate onset of hives, lip swelling, and shortness of breath following the ingestion of a combination cough and cold preparations that contained DM. The patient declined skin testing and underwent a two-step graded oral challenge to DM where she developed hives, nasal and conjunctival congestion following the full dose. Importantly, this patient later tolerated hydrocodone and codeine [6]. The second report describes a 24-year-old female who developed anaphylaxis immediately following ingestion of Romilar® (dextromethorphan hydrobromide). She was positive on skin prick testing to Romilar®, as were 3 of 10 control subjects. She went on to receive an oral challenge to DM syrup and developed generalized erythema, pruritus and hypotension, requiring epinephrine. This patient subsequently tolerated codeine and tramadol [7].

The third report details a case of a 14-year-old female who experienced anaphylaxis following exposure to DM. Although skin prick testing and the Basophil Activation Test (BAT) were negative, the patient developed generalized erythema and hives during drug provocation testing with DM [8].

At our clinic, we initially performed skin prick testing to several drugs as our patient reported ingestion of multi-ingredient products prior to his reactions, making identification of the culprit agent challenging. Owing to inconsistent skin prick testing results, the test was repeated three times. DM was positive in both the first and third testing attempts. This variation in skin prick results may be related to the difference in the number of mast cells present at a body site even within the same limb [10].

Skin prick testing with DM at full concentration was performed on two healthy individuals. Both tests were negative, with no immediate hypersensitivity reactions observed.

We expected that our patient would have a positive skin test to the formulation of Benylin® that contains DM, but his testing result was indeterminate, which could be due to the low concentration of the culprit allergen in the mixture, resulting in a weakly positive reaction on skin testing.

Our case has several diagnostic limitations. The exact brand of Vicks DayQuil Complete® was unavailable. Hypersensitivity to excipients or dyes was considered but could not be tested due to lack of isolated forms. However, the patient had previously tolerated other medications with the same ingredients, such as those in Vicks DayQuil and Tylenol. Basophil activation testing was not pursued due to lack of a validated assay for DM [8] and drug provocation was avoided given the severity of the initial reaction. Skin prick testing was performed using undiluted formulations, which may carry a risk of irritant responses.

Anaphylaxis may result from classical IgE-mediated mechanisms or through non-IgE pathways such as direct mast cell activation [11]. One such pathway involves activation of the MRGPRX2 receptor, a G protein–coupled receptor expressed on mast cells. MRGPRX2-mediated activation has been implicated in hypersensitivity reactions to several small-molecule drugs, including opioids such as morphine, codeine and DM [12].

While clinical features of MRGPRX2-mediated reactions can mimic IgE-mediated anaphylaxis, certain features may favor the former—such as reactions occurring on first exposure, the need for higher doses to trigger symptoms, and shorter symptom duration [13].

The diagnostic distinction can be difficult, as both pathways may produce similar clinical presentations. Additionally, positive responses on skin testing can occur due to direct mast cell activation, complicating interpretation. As such, the positive skin test to DM observed in our patient does not definitively indicate an IgE-mediated mechanism, and alternative pathways, including MRGPRX2 involvement, should be considered in the differential diagnosis.

A recently published pediatric report of DM-induced anaphylaxis, confirmed by drug provocation despite negative skin prick and BAT results, further underscores the potential involvement of non-IgE mechanisms and the diagnostic limitations of current testing methods [8]. These observations highlight the need for greater awareness and further research into the clinical implications of MRGPRX2-mediated hypersensitivity.

Although DM is structurally related to phenanthrene opioids, cross-reactivity has not been clearly demonstrated [7, 14]. Therefore, the patient was advised to avoid DM specifically, rather than all chemically related opioids. However, as DM is structurally related to pholcodine, a cough medication used in Europe for many years that was implicated in perioperative anaphylaxis through cross-sensitization with neuromuscular blocking agents, the potential for cross-reactivity within this chemical class cannot be excluded [15].

Skin testing to phenanthrene opioids like morphine and codeine is not performed due to the irritant effect of these agents and the high risk of false positive results [16]. Nor did we offer challenge doses to these drugs, although such evaluation may have provided additional insight into potential cross-allergenicity. This represents a limitation of our study and highlights the need for further investigation into immunologic cross-reactivity among structurally related compounds.

## Conclusion

We report a rare case of anaphylaxis to a multi-ingredient cough syrup, with subsequent skin prick testing being positive for DM only. Our case highlights the utility of skin prick testing to suspected culprits to identify the allergen. Anaphylaxis to DM is rare and possible mechanisms include IgE-mediated hypersensitivity or direct mast cell activation. Given the widespread use of DM, clinicians should be aware of this potential reaction.

## Data Availability

No datasets were generated or analysed during the current study.

## Abbreviations

AGEP: Acute Generalized Exanthematous Pustulosis
BES: Benylin® Extra Strength All-In-One Syrup
DM: Dextromethorphan
DSL: Dayquil® Sinus Liquicaps
BAT: Basophil activation test
